# Alveolar T-helper 17 responses to *streptococcus pneumoniae* are preserved in ART-untreated and treated HIV-infected Malawian adults

**DOI:** 10.1016/j.jinf.2017.10.013

**Published:** 2018-02

**Authors:** Chikondi Peno, Dominic H. Banda, Ndaru Jambo, Anstead M. Kankwatira, Rose D. Malamba, Theresa J. Allain, Daniela M. Ferreira, Robert S. Heyderman, David G. Russell, Henry C. Mwandumba, Kondwani C. Jambo

**Affiliations:** a Malawi-Liverpool-Wellcome Trust Clinical Research Programme, University of Malawi College of Medicine, Blantyre, Malawi; b Department of Medicine, University of Malawi College of Medicine, Blantyre, Malawi; c Department of Clinical Sciences, Liverpool School of Tropical Medicine, Liverpool, UK; d Division of Infection & Immunity, University College, London, UK; e Department of Microbiology and Immunology, College of Veterinary Medicine, Cornell University, Ithaca, New York, USA

**Keywords:** *Streptococcus pneumoniae*, ART, HIV, T helper 17, Pneumonia, Lung, BAL fluid

## Abstract

**Objective:**

We explored if HIV infection is associated with impaired T-Helper 17 responses against *Streptococcus pneumoniae* in the lung.

**Methods:**

We recruited 30 HIV-uninfected healthy controls, 23 asymptomatic HIV-infected adults not on ART, and 40 asymptomatic HIV-infected adults on ART (Median time 3.5yrs), in whom we collected bronchoalveolar lavage fluid. We measured alveolar CD4^+^ T cell immune responses following stimulation with pneumococcal cell culture supernatant using flow cytometry-based intracellular cytokine staining.

**Results:**

We found that the proportion of alveolar CD4^+^ T cells producing IL-17A following stimulation with pneumococcal cell culture supernatant (CCS) was similar between HIV-uninfected controls and ART-naïve HIV-infected adults (0.10% vs. 0.14%; p = 0.9273). In contrast, the proportion and relative absolute counts of CD4^+^ T cells producing IL-17A in response to pneumococcal CCS were higher in ART-treated HIV-infected adults compared HIV-uninfected controls (0.22% vs. 0.10%, p = 0.0166; 5420 vs. 1902 cells/100 ml BAL fluid; p = 0.0519). The increase in relative absolute numbers of IL-17A-producing alveolar CD4^+^ T cells in ART-treated individuals was not correlated with the peripheral blood CD4^+^ T cell count (r=–0.1876, p = 0.1785).

**Conclusion:**

Alveolar Th17 responses against *S. pneumoniae* are preserved in HIV-infected adults. This suggests that there are other alternative mechanisms that are altered in HIV-infected individuals that render them more susceptible to pneumococcal pneumonia.

## Highlights

• Pneumococcal-specific Th17 responses in HIV-infected adults are preserved.
• The frequency of pneumococcal-specific Th17 cells is increased in ART-treated HIV-infected adults.
• Depletion of pneumococcal-specific Th17 cells is unlikely the reason behind the increased susceptibility to pneumonia in HIV-infected adults.

## Introduction

Invasive pneumococcal disease (IPD), in the form of pneumonia and bacteraemia is a leading cause of mortality worldwide.^1^, ^2^ HIV-infected adults are 60 times more likely to suffer IPD than age-matched HIV-negative persons.^3^, ^4^, ^5^ Initiation of antiretroviral therapy (ART) has led to a reduction in the incidence of IPD in HIV-infected individuals.^4^ Nevertheless, the risk of pneumococcal pneumonia is still 30-fold higher in HIV-infected persons on ART compared to HIV-uninfected individuals.^6^, ^7^ It is not clear what factors are behind the persistent high risk of pneumococcal pneumonia in HIV-infected individuals on ART.

We have previously shown that IFN-γ- and TNF-producing alveolar CD4^+^ T cell responses to *Streptococcus pneumoniae* are maintained in asymptomatic chronic HIV-infected individuals.^8^ This suggested that the increased risk to pneumococcal pneumonia in these individuals might not be due to depletion of these important CD4^+^ T cell subsets in the alveoli. Recently, IL-17A-producing CD4^+^ T cells in the lung have been shown to be critical in conferring protection in murine models of pneumococcal lung infection.^9^, ^10^ In humans, our data from an experimental pneumococcal nasal carriage model showed that pneumococcal carriage leads to increased frequency of pneumococcal-specific Th17 cells in the lung, and that alveolar macrophages exhibited enhanced killing of opsonised pneumococci upon stimulation with recombinant human IL-17A.^11^ Furthermore, children that are prone to acute otitis media have been shown to have reduced proliferation and differentiation of pneumococcal-specific IL-17A-producing CD4^+^ T cells in peripheral blood compared with non-infection prone children.^12^ Taken together, these studies suggest that Th17 cells may play an important role in conferring protection against mucosal infection in adults. However, the link between pneumococcal-specific Th17 immunity and increased risk of pneumococcal pneumonia in HIV-infected individuals has not yet been substantiated.

We therefore explored the possibility that Th17 responses against *S. pneumoniae* in the lung are impaired in HIV-infected adults and are not reconstituted with ART. We determined the proportion of alveolar CD4^+^ T cells producing IL-17A, TNF and IFN-γ after stimulation with pneumococcal cell culture supernatant (CCS), in HIV-uninfected controls compared to untreated or ART-treated asymptomatic HIV-infected adults.

## Results

### Participant characteristics

We recruited 30 HIV-uninfected healthy controls (median age [range] 39[18–44]; male:female, 22:8) and 63 asymptomatic HIV-infected adults (median age [range] 32[20–46]; male:female,16:47), 23 of whom were ART-naive and 40 were receiving ART (median time on ART [range] 3.5yrs [0.7–9.8]). Two-thirds (30/40) of the ART-treated participants were receiving stavudine/lamivudine/nevirapine therapy, while one-third (10/40) were on tenofovir/lamivudine/efavirenz therapy according to national treatment guidelines. The peripheral blood CD4 count of the ART-naïve HIV-infected adults was lower than that of HIV-uninfected controls (399 vs. 818 cells/µl, p < 0.0001). Similarly, the peripheral blood CD4 count of the ART-treated HIV-infected adults was lower than that of HIV-uninfected controls (450 vs. 818 cells/µl, p < 0.0001). However, the peripheral blood CD4 count of the ART-naïve HIV-infected adults was not statistically significantly different from ART-treated HIV-infected adults, but the viral load was lower in the ART-treated HIV-infected adults, with 85% (34/40) of the individuals having a plasma HIV viral load of <150 copies/ml. The main characteristics of the participants are summarized in Table 1.

**Table 1:** Demographics of study participants.

	HIV-uninfected (n = 30)	HIV-infected ART-naive (n = 23)	HIV-infected on ART (n = 40)
**Age (years), median (range)**	29(18–44)	30(20–44)	34(20–46)
**Sex (M:F)**	22:8	8:15	8:32
**BAL lymphocyte count (cells/100 ml BAL fluid), median (IQR)**	5.8x10^6^(3.6–11.7x10^6^)	8.7x10^6^(4.9–17.5x10^6^)	8.3x10^6^(5.2–16.2x10^6^)
**BAL CD4 count (cells/100 ml BAL fluid), median (IQR)**	2.9x10^6^(1.5–5.9x10^6^)	2.8x10^6^(1.9–5.2x10^6^)	2.3x10^6^(1.5–5.0x10^6^)
**Peripheral blood CD4 count (cells/µl), median (IQR)**	818(668–964)	399(244–556)	450(321–640)
**Plasma viral load (copies/ml), median (IQR)**	N/A	33944(3364–289733)	a4476(607–198088)
**Years on cART, median (range)**	N/A	N/A	3.5(0.7–9.8)

IQR,  interquartile range; N/A,  not applicable.

a This is for 6 out of 40 ART-treated HIV-infected individuals (the remaining 34 had plasma HIV viral load of <150 copies/ml, the lower limit of detection of the assay).

### Alveolar CD4+ T cell cytokine responses against *S. pneumoniae* are preserved in HIV-infected adults

**Figure 1:**
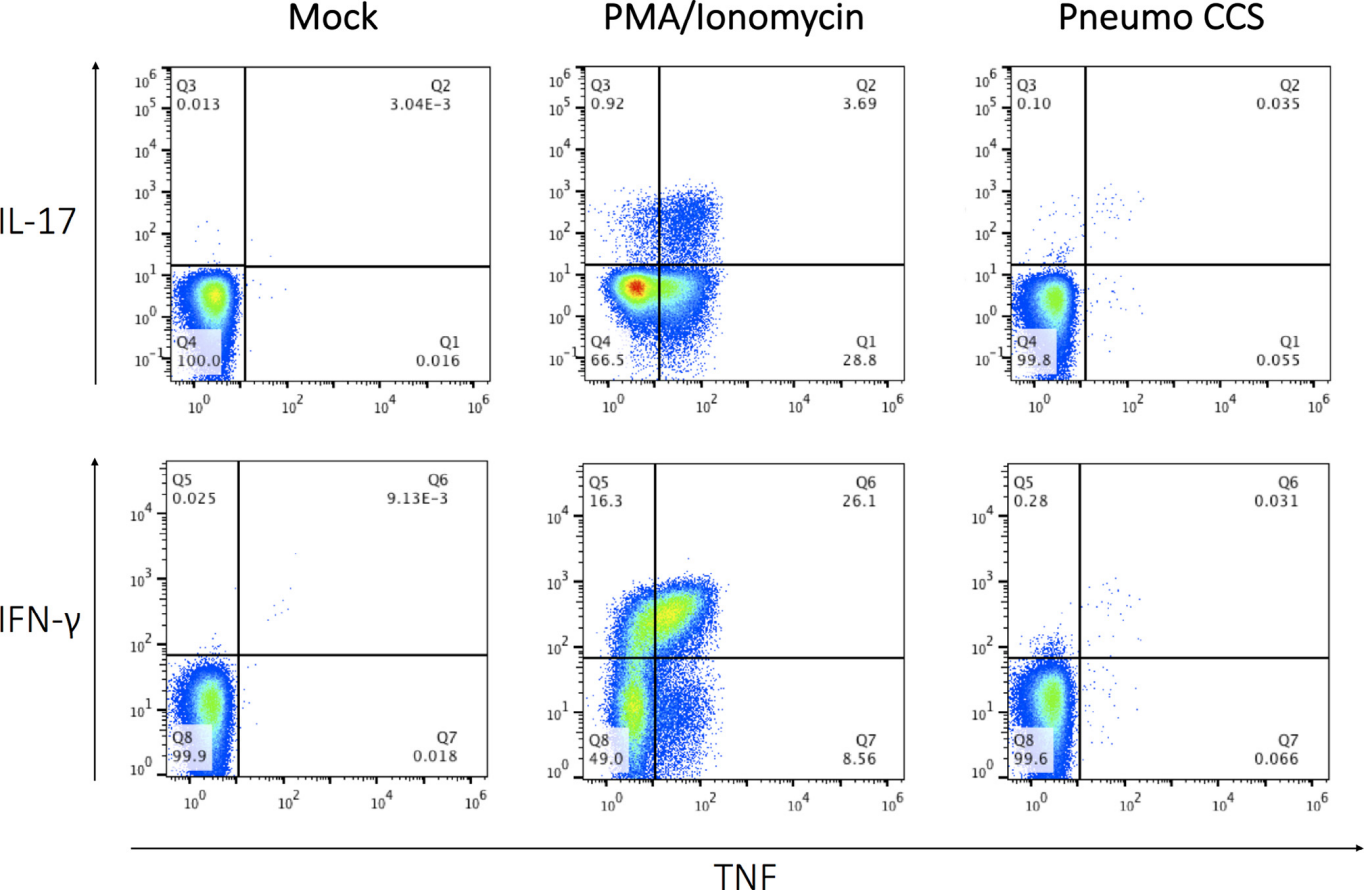
Representative flow cytometry dot plots showing pneumococcal-specific alveolar CD4^+^ T cell responses in BAL fluid from a healthy HIV-uninfected adult**.** Non-adherent bronchoalveolar lavage (BAL) cells were stimulated overnight with PMA/Ionomycin and pneumococcal cell culture supernatant (Pneumo CCS) for 18 hours and CD4^+^ T cell responses were measured by intracellular cytokine staining. The dot plots were obtained by gating on singlets, lymphocytes, live cells, CD3^+^ cells, CD4^+^ cells and combination of three cytokines. The plots show the frequency of interferon-γ (IFN-γ), Tumor Necrosis Factor (TNF) and/or IL-17A-producing CD4^+^ T cells in BAL, in non-stimulated negative control and cells stimulated with PMA/Ionomycin (Positive control), and pneumococcal cell supernatant antigens.

**Figure 2:**
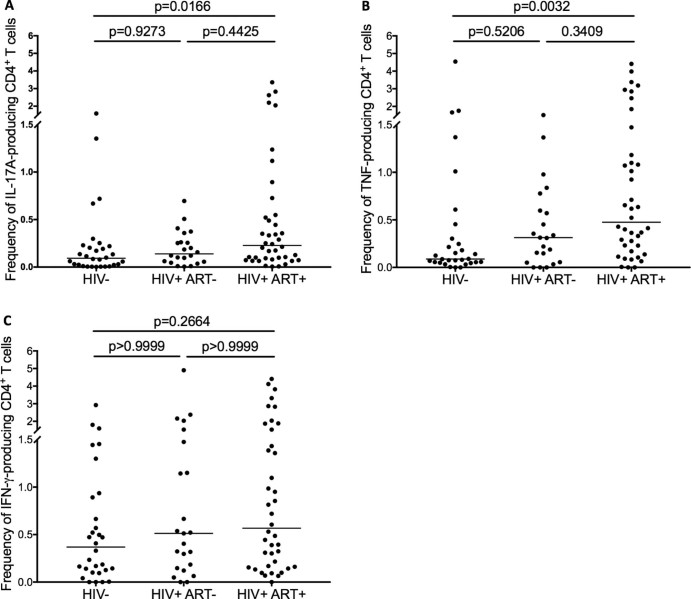
Proportion of cytokine-producing alveolar CD4^+^ T cells after stimulation with pneumococcal cell culture supernatant**.** BAL cells were stimulated with pneumococcal cell culture supernatant and cytokine-producing cells were enumerated by flow cytometry-based intracellular cytokine staining. Each data point represents the proportion of A) IL17A-, B) TNF- and C) IFN-γ-producing alveolar CD4^+^ T cells after subtracting background responses obtained from the non-stimulated controls. The horizontal bars represent median. Data were analyzed using Kruskal-Wallis test and Dunn's multiple comparisons test (HIV-, n = 30; HIV+ ART naive, n = 22; HIV+ ART+, n = 40). Pneumo CCS, pneumococcal cell culture supernatant.

Flow cytometry-based intracellular cytokine staining for IL-17A, TNF and IFN-γ was used to detect CD4^+^ T cell responses following stimulation of BAL cells with pneumococcal cell culture supernatant (Fig. 1). We found comparable proportions of IL-17A-producing alveolar CD4^+^ T cells between ART-naïve HIV-infected adults and HIV-uninfected controls (Median 0.14% [Interquartile range 0.05–0.30] vs. 0.10% [0.02–0.20]; p = 0.9273) (Fig. 2A). Similarly, there were no significant differences in the proportions of TNF- and IFN-γ-producing alveolar CD4^+^ T cells between ART-naïve HIV-infected adults and HIV-uninfected controls (0.31% [0.05–0.78] vs. 0.10%[0.05–0.34]; p = 0.5206 and 0.51% [0.15–1.48] vs. 0.37%[0.12–0.90]; p > 0.9999, respectively) (Fig. 2B and 2C).

### ART is associated with increased proportion of IL-17A/ TNF-producing alveolar CD4^+^ T cells against *S. pneumoniae* in HIV infected adults

We then investigated the impact of ART on IL-17A, TNF and IFN-γ-producing alveolar CD4^+^ T cells, by stimulating BAL cells obtained from ART-treated HIV-infected adults with pneumococcal CCS and determining the proportions of the responding cells using flow cytometry-based intracellular cytokine staining. We found that the proportion of IL-17A-producing alveolar CD4^+^ T cells was higher in ART-treated HIV-infected adults compared to HIV-uninfected individuals (Median 0.22% [Interquartile range 0.08–0.50] vs. 0.10%[0.02–0.20]; p = 0.0166) (Fig. 2A). Similarly, the proportion of TNF-producing alveolar CD4^+^ T cells was higher in ART-treated HIV-infected adults compared to HIV-uninfected individuals (0.51% [0.18–1.16] vs. 0.10%[0.05–0.34]; p = 0.0032) (Fig. 2B). In contrast, there was no statistically significant difference in proportion of IFN-γ-producing alveolar CD4^+^ T cells between ART-treated HIV-infected adults and HIV-uninfected individuals (0.57% [0.16–1.51] vs. 0.37%[0.12–0.90]; p = 0.2664) (Fig. 2C).

### No association between the absolute numbers of IL-17A-producing alveolar CD4^+^ T cells and total CD4^+^ T cell count in BAL fluid in HIV-infected adults

**Figure 3:**
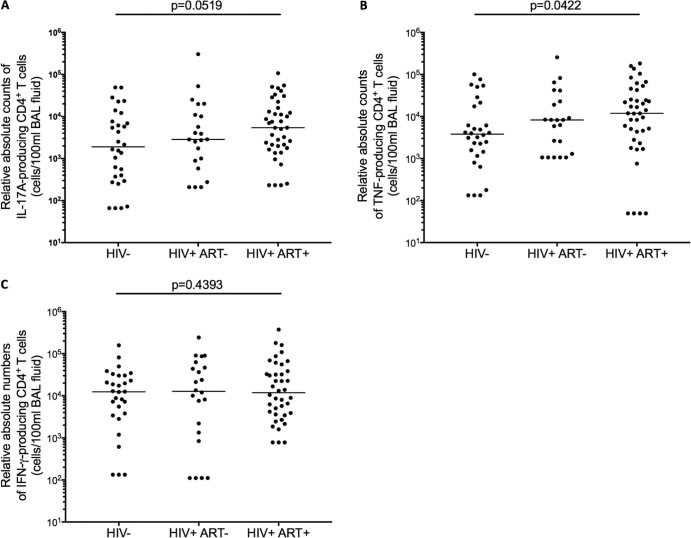
Relative absolute counts of cytokine-producing alveolar CD4^+^ T cells after stimulation with pneumococcal cell culture supernatant. Using the proportions of IL-17A/TNF/IFN-γ-producing alveolar CD4^+^ T cells responding to stimulation with pneumococcal cell culture supernatant, we derived their relative absolute counts from the total count of alveolar CD4^+^ T cells. Each data point represents the relative absolute count of A) IL17A-, B) TNF- and C) IFN-γ-producing alveolar CD4^+^ T cells. The horizontal bars represent median. Data were analysed using Mann Whitney U test (HIV-, n = 30; HIV+ ART naive, n = 22; HIV+ ART+, n = 40).

We then investigated whether the higher frequencies of IL-17A and TNF-producing alveolar CD4^+^ T cells were correlated with ART-driven reconstitution of total CD4^+^ T cells. First, we determined whether the increase in IL-17A/TNF-producing alveolar CD4^+^ T cells was not only qualitative but also quantitative, by comparing the relative absolute counts of the responding T helper cells among the groups. Using the proportions of IL-17A/TNF/IFN-γ-producing alveolar CD4^+^ T cells in Fig. 2, we derived their relative absolute counts from the total count of alveolar CD4^+^ T cells (Table 1). We found that the relative absolute counts of IL-17A-producing alveolar CD4^+^ T cells was higher in ART-treated HIV-infected adults compared to HIV-uninfected individuals (Median 5420 cells/100 ml BAL fluid [Interquartile range 1829–15286] vs. 1902 [67–8758]; p = 0.0519) (Fig. 3A). Similarly, the relative absolute counts of TNF-producing alveolar CD4^+^ T cells was higher in ART-treated HIV-infected adults compared to HIV-uninfected individuals (11918 cells/100ml [3182–38405] vs. 3808 [1376–18461]; p = 0.0422) (Fig. 3B). In contrast, there was no statistically significant difference in relative absolute counts of IFN-γ-producing alveolar CD4^+^ T cells between ART-treated HIV-infected adults and HIV-uninfected individuals (11837 cells/100ml [3680–37017] vs. 12415[3261–30270]; p = 0.4393) (Fig. 3C). Furthermore, there was no association between plasma viral load and the alveolar CD4^+^ T cell responses in HIV-infected individuals (p = 0.5644, r = 0.1371).

Lastly, we determined whether there was an association between the relative absolute counts of IL-17A/TNF-producing alveolar CD4^+^ T cells and peripheral blood CD4 count in the ART-treated and untreated HIV-infected adults. We did not find any statistically significant correlation between the relative absolute counts of IL-17A/TNF-producing alveolar CD4^+^ T cells with peripheral blood CD4 count (IL-17A, r=–0.1876, p = 0.1785; TNF, r=–0.1689, p = 0.2267).

### HIV infection is associated with minimal disruption of polyfunctional responses of cytokine-producing alveolar CD4^+^ T cells against *S. pneumoniae*

**Figure 4:**
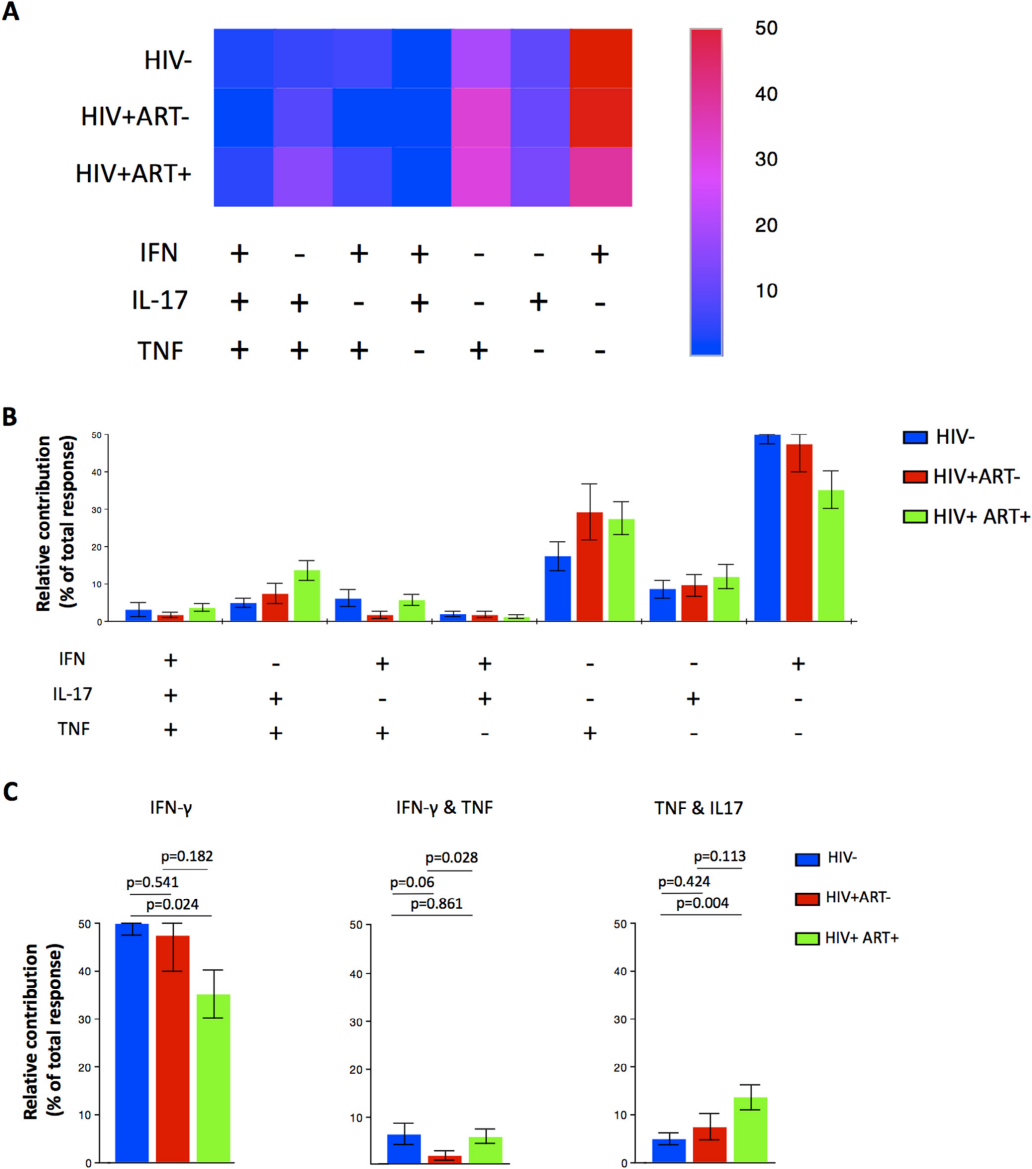
Functional profile of alveolar CD4 T cells after stimulation with pneumococcal cell culture supernatant. Cells were analysed by flow cytometry for IFN-γ, TNF, and IL-17A production by the CD4+ T cell population. Data are shown as relative distribution of IFN-γ, TNF, and IL-17A producing CD4^+^ T cell subsets within the total response. A) Heat map showing the mean distribution across subjects of different antigen-specific cytokine-producing CD4^+^ T cell subsets within the total response. B) Bar charts represent the mean and standard error of the mean (SEM) of the contribution of the indicated subset (x-axis) towards the total antigen-specific CD4^+^ T cell response against the indicated participant groups (color coded as shown). C) Relative distribution of subsets within the total response which are statistically significant among the study groups. Data were analysed using using student t test. (HIV-, n = 30; HIV+ ART naive, n = 22; HIV+ ART+, n = 40).

Next, we investigated whether there were any functional differences in the Th1 and Th17 responses against *S. pneumoniae* among the HIV-uninfected controls, ART-naïve and ART-treated HIV-infected adults. We determined the impact of HIV and ART on the Th1 and Th17 response as previously described.^13^ The analysis allowed identification of 7 different functional subsets namely; IFN-γ^+^TNF^+^IL17^+^, IFN-γ^+^TNF^+^IL17^-^, IFN-γ^+^TNF^-^IL17^+^, IFN-γ^-^TNF^+^IL17^+^, IFN-γ^+^TNF^-^IL17^-^, IFN-γ^-^TNF^+^IL17^-^ and IFN-γ^-^TNF^-^IL17^+^ cells. We found that there were no differences in the composition of total CD4^+^ T cell responses against pneumococcal antigens between the HIV-uninfected controls and ART-naive HIV-infected adults, but there were differences between ART-treated HIV-infected adults and HIV-uninfected controls or ART-naïve HIV-infected adults (Fig. 4A). Specifically, the relative contribution of pneumococcal-specific IFN-γ^+^ CD4^+^ T cells single producers in the total response was lower in ART-treated HIV-infected adults compared to HIV-uninfected controls (35% vs. 50%, p = 0.024) (Fig. 4B and 4C). The relative contribution of pneumococcal-specific IFN-γ^+^TNF^+^CD4^+^ T cells double producers in the total response was lower in ART-naive HIV-infected adults compared to ART-treated HIV-infected adults (2% vs. 6%, p = 0.028) (Fig. 4B and 4C). The relative contribution of pneumococcal-specific IL17^+^TNF^+^CD4^+^ T cells double producers in the total response was higher in ART-treated HIV-infected adults compared to HIV-uninfected individuals (15% vs. 5%, p = 0.004) (Fig. 4B and 4C). These findings suggest that pneumococcal-specific polyfunctional alveolar CD4^+^ T cell responses are minimally impacted by HIV infection.

## Discussion

We explored whether Th17 responses against *S. pneumoniae* in the lung are impaired in HIV-infected individuals. We found preserved IL-17A/TNF/IFN-γ CD4^+^ T cell immune responses in ART-naïve HIV-infected adults, increased proportions and relative absolute counts of IL-17A/TNF-producing CD4^+^ T cells in BAL cells from ART-treated HIV-infected adults following stimulation with pneumococcal CCS.

This lack of perturbation in the CD4^+^ T cell responses of the lung against *S. pneumoniae* supports our previous findings that TNF-/IFN-γ-producing alveolar CD4^+^ T cell responses are not impaired in asymptomatic HIV-infected adults.^8^ This mucosal response appears compartmentalised^13^, ^14^ as we have previously shown that pneumococcal-specific CD4^+^ T cell responses from the systemic circulation are impaired in HIV-infected adults^15^ and not fully reconstituted following ART.^16^ However, even though the IL-17A/TNF/IFN-γ producing alveolar CD4^+^ T cell responses against *S. pneumoniae* in HIV-infected adults are preserved, there are other immune processes in the alveolar space that have been shown to be compromised in ART-naïve HIV-infected adults, which can lead to an increased risk to lower respiratory tract infections. HIV infected adults have been shown to have impaired alveolar macrophage proteolytic function,^17^ poor lung IgG opsonic function,^18^ dysregulated alveolar cytokine networks^19^ and lymphocytic alveolitis,^20^, ^21^ which could mask the potential beneficial effects of the local alveolar CD4^+^ T cell responses against *S. pneumoniae.*

Interestingly, the proportions of IL-17A and TNF-producing alveolar CD4^+^ T cells responding to pneumococcal stimulation were higher in ART-treated HIV-infected adults compared to HIV-uninfected counterparts. Consistent with observations that Th17 cells are among the major producers of TNF,^22^, ^23^ there was a strong association between the IL-17A and TNF-producing alveolar CD4^+^ T cells (data not shown). However, we found no association between the relative absolute counts of IL-17A/TNF-producing CD4^+^ T cells with peripheral blood CD4^+^ T cell count. Furthermore, the absolute counts of IL-17A/TNF-producing CD4^+^ T cells were higher in the ART-treated HIV-infected adults compared to the HIV-uninfected controls. These findings suggest that the increase in Th17 responses against *S. pneumoniae* in ART-treated individuals is unlikely due to general reconstitution of CD4^+^ T cells. On the other hand, this might be an immune reconstitution inflammatory syndrome (IRIS)-like phenomenon, which occurs in some individuals on ART and is associated with an overwhelming response to a previously acquired infection.^24^, ^25^

Pneumococcal carriage in humans is immunising and leads to increased frequency of pneumococcal-specific IL17-producing CD4^+^ T cells in the lung.^11^ The level of pneumococcal exposure is relatively high in Malawi, with pneumococcal carriage rates between 15–45%^15^, ^16^, ^26^ in adults and 45–60% in children.^27^, ^28^ The preservation of the IL-17A/TNF/IFN-γ producing alveolar CD4^+^ T cell responses against *S. pneumoniae* in HIV-infected adults may be due to this high degree of mucosal pneumococcal exposure. Previous studies from Malawi have reported higher nasopharyngeal pneumococcal carriage rates in ART-treated HIV-infected adults (41–52%) compared to HIV-uninfected individuals (13–14%) and untreated HIV-infected adults (19–27%).^15^, ^16^ It is therefore plausible that the increased proportions of IL-17A and TNF-producing alveolar CD4^+^ T cells responding to pneumococcal CCS in ART-treated HIV-infected adults might be a reflection of the increased mucosal pneumococcal exposure.

The potential limitation of our study is that the two HIV-infected groups (ART-naïve and treated) were female biased compared to the HIV-uninfected group, however, analysis of the alveolar CD4^+^ T cell responses based on gender did not show any statistically significant differences among the groups (data not shown). The other limitation of the study is that we measured the CD4^+^ T cell responses against *S. pneumoniae in vitro,* which might not accurately represent the behaviour of these cells *in vivo*. However, this approach allowed us to ascertain the presence or absence of pneumococcal-specific alveolar Th17 cells in HIV-infected adults capable of producing IL-17A, TNF and IFN-γ upon stimulation with pneumococcal antigens.

In conclusion, we have found that alveolar Th17 responses against *S. pneumonia* are preserved in HIV-infected adults. This suggests that there might be other alternative mechanisms rendering HIV-infected individuals more susceptible to pneumococcal pneumonia. Serotype-independent T cell-based pneumococcal vaccines that focus solely on inducing a robust Th17 response may not promote clearance of lung infection and prevent pneumonia in HIV-infected adults, up and above naturally-acquired immunity in the alveolar space, since these individuals already posses robust pneumococcal-specific alveolar Th17 cells. We recommend that future studies should examine these Th17 responses in individuals with pneumococcal pneumonia to ascertain their role in humans.

## Methods

### Subjects

The study was conducted at the Queen Elizabeth Central Hospital, a large teaching hospital in Blantyre, Malawi. Participants were recruited from the hospital's voluntary counselling and testing (VCT) and ART clinics. They were healthy, asymptomatic adults (≥18yrs), comprising HIV-1-uninfected and HIV-1-infected volunteers with no clinical evidence of active disease, willing to undergo bronchoscopy and bronchoalveolar lavage (BAL) for research purposes.^29^ HIV testing was performed on whole blood using two commercial point-of-care rapid HIV test kits, Determine HIV 1/2 kit (Abbott Diagnostic Division) and Unigold HIV 1/2 kit (Trinity Biotech Inc.). A participant was considered HIV-uninfected if the test was negative by both kits or HIV-infected if the test was positive by both kits. If Determine and Unigold results were discordant, a third rapid test using Bioline HIV 1/2 kit (Standard Diagnostics Inc.) was performed to resolve the discordance. HIV infected individuals were commenced on ART according to the Malawi standardised national protocols: WHO stage III/IV disease or CD4 count of ≤350 (the cut-off used in Malawi at the time of study recruitment). None of the participants had received pneumococcal vaccination prior to joining the study. The research ethics committee of the Malawi College of Medicine approved the study and all participants provided written-informed consent.

HIV-1-infected participants were divided into two subgroups based on whether they were on ART or not at the time of recruitment. First line ART consisted of stavudine, lamivudine, and nevirapine, or tenofovir, lamivudine, and efavirenz (following a change in the national guidelines in 2011). Exclusion criteria for the study were: current or history of smoking, prior history of pneumococcal disease or pulmonary TB, use of immunosuppressive drugs, severe anaemia (Hb <8g/dl) and known or suspected pregnancy. Peripheral blood CD4^+^ T cell counts were measured in all participants while HIV plasma viral load measurements were performed in HIV-infected participants only. HIV-1 RNA levels in blood plasma were measured using the Abbot Real-Time HIV-1 assay with a lower limit of detection of 150 copies/mL (Abbott Molecular, Germany), per the manufacturer's instructions.

### Collection and processing of BAL fluid and blood samples

BAL fluid samples were collected at bronchoscopy and processed as previously described.^8^, ^30^ Non adherent cells, which consist mainly of lymphocytes, were enriched by adherence of macrophages to plastic,^17^ and were used to assess the frequency of antigen-specific CD4^+^ T cells. Peripheral blood samples were also collected from participants. Plasma samples were used for measurements of HIV viral load.

### Intracellular cytokine staining

Antigen-specific CD4^+^ T cell responses were measured as previously described.^8^ In brief, non-adherent BAL cells (0.5x10^6^cells/well) suspended in 200 µl of complete media were cultured in 96 well plates and stimulated with pneumococcal cell culture supernatant (8 µg/ml) (prepared from a standard encapsulated type 2 (D39) *S. pneumoniae* strain as previously described^16^, ^31^). The cell culture supernatant has been previously utilised to probe pneumococcal-specific T cell immunity,^8^, ^15^, ^16^, ^31^ is rich in pneumococcal surface proteins and pneumolysin.^31^ Cells were stained with Violet Viability dye (LIVE/DEAD® Fixable Dead Cell Stain kit, Invitrogen, UK), surface markers (anti-CD3 PE-Cy5, anti-CD4 APC-H7 and anti-CD8 PE-Cy7 antibodies, all from BD Bioscience, UK), and intracellular markers (anti-IFN-γ APC, anti-tumour necrosis factor (TNF) Alexa Fluor 488 and anti-IL17 PE antibodies, all from BD Bioscience, UK). Approximately 50,000 events were acquired in the CD4^+^ gate using a CyAn ADP 9-colour flow cytometer (Beckman Coulter, USA). Flow cytometry data were analysed using FlowJo software (TreeStar, USA).

### Statistical analyses

Statistical analyses and graphical presentation were performed using GraphPad Prism 5 (GraphPad Software, USA) or PESTLE 1.7 and SPICE 5.3 (both NIAID, USA). The programs PESTLE and SPICE were kindly provided by Mario Roederer, Vaccine Research Center, NIAID, NIH. Flow cytometry data were analysed using Kruskal-Wallis test and Dunn's multiple comparisons test. Results are given as median and interquartile range (IQR). Correlations were measured using Spearman's test. Differences were considered statistically significant when p < 0.05.

## Conflict of interest

All authors declare no competing interests. The information contained in this manuscript has previously not been presented at any meeting and has not been submitted for publication elsewhere.
